# A qualitative study of parental associations and beliefs regarding the HPV vaccination for Dutch boys

**DOI:** 10.1186/s12889-022-13605-y

**Published:** 2022-06-14

**Authors:** Judith R. Venderbos, Renske Eilers, Hein de Vries, Kim van Zoonen

**Affiliations:** 1 Faculty of Health, Medicine and life Sciences, Department of Health Promotion, Maastricht University, Maastricht, The Netherlands; 2 Centre for Infectious Disease Control, National Institute for Public Health and the Environment, Postbus 1, 3720 MA Bilthoven, The Netherlands

**Keywords:** Boys, HPV vaccination, Information needs, National immunization program, Parental beliefs

## Abstract

**Background:**

Human Papillomavirus (HPV) occurs in 80% of the sexually active population and may cause certain types of cancers among men and women. Vaccination against HPV could prevent cancers associated with HPV. The Dutch National Immunization Program (NIP) only includes HPV vaccination for girls, but the HPV vaccination for boys will be implemented in 2022. Little is known about the awareness of parents and their attitudes regarding the vaccination for boys. However, these concepts might influence the intention to vaccinate one’s child. Gaining insight in these factors could lead to more effective communication strategies.

**Methods:**

This qualitative research aimed to explore parental associations and beliefs regarding the HPV vaccination for boys. In total, 16 interviews were conducted with parents. Topics discussed were primary associations with vaccinations, associations with HPV-vaccination and information needs regarding the HPV vaccination for boys.

**Results:**

Most parents were unaware about HPV infections in boys as well as the possibility to vaccinate their sons. Furthermore, after hearing about the vaccine parents reported uncertainties about anticipated adverse effects of the vaccine. Other themes that emerged were difficulties with the proposed age at which boys would be offered the vaccination and low risk perception of the virus.

**Conclusion:**

Public campaigns regarding (the HPV) vaccination should improve (parental) awareness about the virus and the vaccination, and increase knowledge. Moreover, it should address concerns regarding vaccination and be transparent about decision making. For example, a rationale why HPV vaccination is needed for boys who turn 10 years and its advantage to reducing risks for boys to contract HPV related cancers should be provided.

**Supplementary Information:**

The online version contains supplementary material available at 10.1186/s12889-022-13605-y.

## Background

Worldwide, Human Papillomavirus (HPV) infections occur in 80% of the sexually active population [1]. Although the majority of these infections are cleared up without severe symptoms and/or treatment, some high-risk types of the virus are associated with several types of cancer among men and women [2, 3]. Cervical cancer is the most common, as 99% of the cervical cancers are linked to a HPV infection, however penile, vulvar, anal, vaginal as well as head and neck cancers are also associated with HPV infections [4–8]. According to the Dutch Cancer Institute (NKI), annually 1084 women and 316 men in the Netherlands develop cancer associated with HPV [9]. Worldwide, it is estimated that HPV vaccines protect against HPV types associated with 90% of cervical cancers and 80–95% of other HPV associated cancers [10, 11].

Vaccination against HPV for girls was included in the Dutch National Immunization Program (NIP) in 2010. Recently, the Dutch Health Council advised to also include HPV vaccination for boys in the NIP, this will be implemented in 2022. However, they will not (yet) advice on the type of HPV vaccine that should be used [12]. Therefore, the same vaccine will be used which is currently used for girls (Cervarix©). This vaccine has shown to be more effective in preventing certain cancers in men, such as head and neck cancers, but also seems to protect boys long enough [11]. Inclusion should decrease HPV associated cancers among men and help protect unvaccinated boys and girls through cross-protection [13, 14]. Vaccination against HPV seems most effective when it is administered prior to the first sexual contact. Therefore, it is advised to vaccinate boys and girls in the year they turn ten years old [14]. Previously, girls were vaccinated at the age of twelve/thirteen years old. In accordance with applicable Dutch legislation, the proposed age implies that HPV vaccination uptake is critically dependent upon parental acceptance of the vaccination for their children. Even more so now the age at which the vaccine will be administered has been lowered to the year children turn ten years old. Identifying factors associated with parental beliefs and intentions regarding the HPV vaccination is essential, as these will influence whether the child gets vaccinated or not [15–17].

Several studies have assessed parental attitudes and information preferences towards the HPV vaccination for girls and parental intentions towards HPV vaccination of their daughters [18–22]. Most recent research on the HPV vaccination also include HPV and (parents of) boys [16, 17, 23–26]. Before beliefs and intentions are developed, awareness encompasses a commonly used initial phase in research. Determinants that influence awareness include knowledge, risk perception and cues to action [27]. Increased knowledge could result in either increased or decreased vaccination acceptance. Some studies have shown that HPV vaccine acceptance increases if people obtain a better understanding of HPV and its potential consequences [28, 29]. However, a better understanding of the potential consequences of HPV has sometimes led to stigma concerning the disease. This could potentially decrease HPV vaccine acceptance [28, 30]. Furthermore, perceived severity of HPV infections have shown to increase parental acceptability of vaccines against sexually transmitted infections, when participants themselves or their children were regarded to be at risk of HPV exposure or infection [31]. Lastly, cues to action pertain to situational circumstances which prompt HPV vaccination awareness and willingness towards uptake, such as information material in public campaigns or knowing people who got vaccinated against HPV [29].

Subsequent to awareness, several other factors also influence vaccination behavior (i.e. acceptance and/or uptake), involving attitudinal beliefs, social influence beliefs, and self-efficacy expectations (e.g. beliefs regarding the role of religion) [27]. Attitudinal factors include health beliefs, for instance regarding concerns about promoting sexual activity or anticipated side effects of the HPV vaccination, which have been reasons for more negative attitudes and lower HPV vaccine acceptability and/or uptake [28, 32]. Another type of beliefs are more emotional beliefs, such as anticipated regret which may increase tendencies towards screening and vaccination [33–35]. Social influence beliefs includes both supportive and opposing influences as experienced from social norms, example behaviors and direct support and opposing influences from others. Examples are partner’s positive norm and recommendations from healthcare professionals [36–39]. Other studies also found the importance of social support and the enabling effect on HPV vaccination uptake for their children through influencing (decisional) self-efficacy beliefs [40, 41].

It is to be expected that many Dutch parents may not be aware of the vaccine and/or convinced to accept the vaccination as it is not yet included in the Dutch NIP. Research regarding Dutch parents of boys and HPV vaccination is scarce, except for one recent study identifying four dominant perspectives on HPV vaccination among parents of both girls and boys before the vaccination program for boys started [17]. Examining awareness and motivational factors and the influence these might have on parental beliefs and intentions regarding the HPV vaccination for boys is therefore important, in order to understand motives and feelings of Dutch parents and to guide information campaigns.

Therefore, the aim of the current study is to identify beliefs about vaccination in general, primary associations of parents about HPV vaccination for boys, and perceived advantages and disadvantages of HPV vaccination for boys.

## Methods

### Study aim, design and setting

In order to examine awareness, parental attitudes and information needs a qualitative, cross-sectional design was used.

#### Participants and recruitment

Dutch-speaking parents and/or guardians (hereafter referred to as parents) with a son born in 2010 were included in the study. This year was chosen, because these boys would have been invited to receive the HPV vaccination if it had already been implemented. Moreover, these parents are based in the Netherlands. Personal networks of the researchers have been used to recruit parents. Furthermore, snowballing as well as several social media channels were used to reach the parental sample (e.g. LinkedIn and Facebook). Furthermore, prior to the outbreak of COVID-19 and the closure of Dutch schools, an invitation letter for parents was sent via four primary schools.

#### Data collection tools

Individual in-depth interviews were conducted in April–May 2020 and were semi-structured (see Table 1 for topics). On average an interview lasted 42 minutes and took place at a time preferred by the participants. The interviews were conducted by telephone due to restrictions because of the COVID-19 pandemic.

**Table 1:** Topic guide

Topic	Question
Overall vaccinating	What is the first thing that comes to mind when you hear the word vaccination?
	What do you overall think about vaccination?
	Was your son vaccinated during his earlier childhood?
	Can you elaborate on the decision of whether or not to vaccinate?
	What do people close to you think about vaccination?
	Is vaccination something you discuss with others?
HPV vaccine	What is the first thing that comes to mind when you hear about the HPV vaccine?
	Do you think the vaccine against HPV differs from other vaccinations?
HPV vaccine for boys	Have you ever heard of the HPV vaccine for boys?
	What is the first thing that comes to mind when you hear about the HPV vaccine for boys?
	Why do you think boys could get vaccinated against HPV?
	How do you think an HPV infection is transmitted?
	Do you think there are advantages of the HPV vaccine for boys?
	Do you think there are disadvantages of the HPV vaccine for boys?
	Which people would be in favor of having your son vaccinated against HPV?
	Which people would be against having your son vaccinated against HPV?
	Do you think it is needed that boys will be vaccinated against HPV?
	Do you think your son is at risk of getting HPV later?
	Do you think it is needed that your son will be vaccinated against HPV?
Discussing the HPV vaccine	Would the HPV vaccine for boys be something you would like to discuss with people in your surrounding? With whom? And why?
	Would you discuss the HPV vaccine for your son with your partner?
	Would you discuss the HPV vaccine for boys with your son?
Visualization (equal for all 3 visuals)	What do you think this visualization is about?
	What feelings does this visualization evoke?
	Is there anything you would like to change about the visualization?
	Do you feel you can understand the visualization?

An inductive approach was used to guide the semi-structured topic guide. First, participants were asked to mention primary associations and experiences with overall vaccinating. Second, associations with HPV vaccination for boys and related beliefs were discussed. Third, parents were asked about their information needs and preferences with regards to the vaccination. Fourth, parents were also asked to describe their initial thoughts regarding three proposed visuals about the HPV vaccination for boys to provoke information needs. These visuals (see Additional file 1) were based on previous information materials about HPV vaccination and included general information about the underlying research process (i.e. the development of the vaccine), a visual representation of HPV cells and the physical areas of HPV associated cancers. The first visual was designed by the researchers themselves, whereas the second visual concerned a modified visual used in Dutch communication materials regarding the HPV vaccination for girls [42]. The third visual was based on a graphic in health communication by Cancer Research UK [43]. Lastly, the visuals were sent by email during the interviews.

#### Data analysis

The recorded interviews were anonymously transcribed verbatim. The transcripts were imported to, and coded in, Atlast.ti. The qualitative analysis was done according to the Grounded Theory Approach (GTA), including open coding, thematically coding and ultimately, constructing a first theory [44, 45]. The approach indicates an inductive methodology, as participants narrate their first associations and beliefs about the subject rather than testing a predefined theory [46]. For this reason, the content of the topic guide was not completely predetermined, and emerging themes were analyzed. Two researchers coded the interviews to identify emergent themes, which proceeded in parallel with data collection. Consultation between the two researchers took place in case of discrepancies. The first stage of data analysis was open coding of five transcripts and subsequently, the second coder also open coded three of these transcripts. This led to joint thematic codes which were used to code next five transcripts thematically in the second stage. The second coder also coded three interviews thematically. The third stage involved data analysis in accordance with codes throughout the first two stages. Due to rapidly reached consensus, a third researcher was not involved for additional consultation.

## Results

### Characteristics of the sample

In total, the parental sample concerned 16 parents. An overview of the characteristics of the participants is presented in Table 2. The interviews were conducted with thirteen mothers and three fathers. The average age was 38 years old. Most parents were born in The Netherlands and were highly educated. Only two parents had older daughters who were earlier invited for the HPV vaccine. Overall, 15 parents got their children vaccinated in accordance with recommendations of the NIP.

**Table 2:** Characteristics of the interviewed participants (*N* = 16)

Gender	**Participants (N)**
Female	13
Male	3
Age	**Participants (N)**^**a**^ (average 38)
31–40	12
41–50	2
51–53	1
Country of birth	**Participants (N)**
The Netherlands	13
Other	3
Civil or marital status	**Participants (N)**^**a**^
Married	12
Single	3
Education level	**Participants (N)**^**a**^
Secondary vocational education (MBO)	4
University of applied sciences (HBO)	10
University (WO)	1
Older daughter(s) invited for the HPV vaccine	**Participants (N)**
Yes	2
No	14
Followed the NIP	**Participants (N)**
Yes	15
No	1

^a^Numbers are based on characteristics of 15 participants

### Perceptions about overall vaccination

Parents were invited to reflect on how they perceived vaccination of their child (ren) in general. The 15 parents that adhere to the NIP reported they do so because they are convinced that vaccines protect their child (ren) against severe diseases. Furthermore, some parents mentioned that vaccination is something that ‘should just be done’ or they indicated that they do not think thoroughly about it:

“In my opinion it was not really a big decision, or something. It is more common.” (M1)

“I did not even have to think about it, actually. I think vaccines are just the most normal thing in the world.” (F1)

Yet, three parents indicated they recently have started to question the necessity of childhood vaccines or underlying reasons why these vaccines are offered:

“It used to be more of a habit, but as the children got older, I started to study it a bit more. Now I do have doubts: is vaccinating my child good or bad? I am skeptical, although my children have had all their recommended vaccines.” (F3)

### Primary associations of HPV vaccine for boys

The first responses of parents revealed that, initially all parents associated the HPV vaccine with girls and the protection against cervical cancer. Sometimes parents indicated to be aware of the HPV vaccine for boys but seemed to mistake another vaccine their son received in the past for the HPV vaccine. However, the majority of parents were unaware of the possible existence of the virus among boys, its risks for severe consequences for boys and the availability of the HPV vaccine for boys. For instance, some parents asked the researcher whether HPV could be harmful for boys:

“Can HPV also cause illness in boys, that you know?” (F5)

“In the year he turned nine, he was vaccinated for something. I would say that was for this one [HPV vaccine]. But I doubt that now.” (F12)

Most parents associated the HPV vaccine for boys mainly with preventing the transmission of the virus, as girls would be protected against severe consequences of the virus if boys are vaccinated as well. Some parents mentioned the prevention of certain types of cancer among men. However, this was often only reported after they had searched for more information on internet:

“Maybe something with regards to sexual transmitted diseases, to make sure it will not be transferable? But to be honest, I am not sure, because you can vaccinate against it. So, I am thinking about testicular cancer, prostate cancer. To prevent that, or to reduce the chance, maybe?” (F8)

### HPV vaccine for boys in the NIP

Initially, four parents stated that the HPV vaccination for boys did not differ from other childhood vaccinations offered in the NIP. Consequently, these four parents indicated they would get their son vaccinated against HPV when the vaccine becomes available. However, most parents identified differences when they compared the HPV vaccination for boys with other vaccinations in the NIP in more detail:

“I do have that feeling [that the HPV vaccine is different compared to other vaccines], I do not know why. Maybe because it is newer, maybe it does not exist as long as other vaccines? I do not know, maybe people around me, the reports, the media are more critical? I have no idea. Yet, I have the feeling it differs.” (M2)

Few parents stated they understood the necessity of including the HPV vaccination for boys in the NIP. This was mainly because of the association between HPV and certain types of cancer. Related to this, the necessity of the HPV vaccine for boys could for most parents be substantiated with numerical information. The majority of the parents were undecided about participating in the NIP regarding the HPV vaccination for boys. These parents explained they needed numbers or percentages of HPV associated diseases in boys or the likelihood with which an HPV associated disease could be reduced by the vaccine in boys. This would be conducive concerning making a statement about the necessity of summoning boys for the HPV vaccine.

“Do you have more information about, maybe percentages, about how much [associated diseases] HPV causes among men? And in how many cases can you prevent that with the vaccine?” (F13)

### Considerations with regards to the age at which the HPV vaccination will be provided

Parents took several factors into account when thinking about the most appropriate age for the HPV vaccination for boys. Some parents indicated no apprehensions regarding the proposed age for the HPV vaccination for boys, whereas most parents had certain concerns. Parents associated HPV vaccination with sexual behavior and these parents assumed that the ten-year-old age at which it will be administered has been determined because of primary sexual activity. Yet, parents believed that their son would not need an HPV vaccine as their son would still be too young at the age of ten to be in need for a vaccination against HPV. Furthermore, some parents indicated that their son may not be ready for conversations regarding sex and/or transmissions due to sexual behaviors at the age of ten, making this age period to be inappropriate. It was also suggested that primary schools could contribute to informing and discussing the HPV vaccination. Conversely, some parents feared that conversations about HPV vaccination may encourage sexual behavior:

“For the boys and girls who start very early [with having sex] and have different partners, for that group this would be ideal. Because you do not know where a disease comes from, if you have different bed partners. But that is something I do not support. […] And also, if I am talking with a ten-year-old about a vaccine for a virus associated with having sex, you should not be surprised if he starts having sex within two years.” (F3)

### Risk perceptions about HPV among boys

Most parents were unaware of the occurrence of HPV among boys. Moreover, a common theme that emerged concerned the low risk perception of HPV for boys compared to other infectious diseases for which there are vaccines in the NIP, as the transferability of other viruses would be quicker and/or unnoticed. Subsequently, some perceived their children to be at lower risks with regards to HPV:

“This [cancer associated with HPV] is a disease you get if you have a lot of sex. But other diseases, such as mumps or rubella, are contagious in a different way. If you get that, you did not ask for it. I am not saying with this type of cancer, that you ask for it, not at all. But you can make the chance smaller by behaving differently.” (F11)

Lower risk perceptions were related to both upbringing and religious beliefs. Four parents expressed that in their child’s upbringing and/or Islamic or Christian religion, sexual intercourse is not something intended to share with more than one partner. Furthermore, if religion played a role in upbringing, they also stated that sexual intercourse was something that should preferably be undertaken after a marriage (i.e. at a later age). Parents stated that this would reduce the risk of getting HPV:

“Anyway, within our religion, we assume, or at least, that is how we educate our children, that there should only be sexual contact if you are married. That means that you hope they have one partner. That is why I wonder: is it likely that the virus will occur?” (F2)

### Perceived advantages and disadvantages of HPV vaccination for boys

Most parents addressed the cross-protection against cervical cancer for girls as the main advantage of vaccinating boys against HPV. Adding to this, some parents mentioned the protection against certain types of cancer among boys as advantage of the HPV vaccine, as they perceived cervical cancer to be severe:

“I think it is a good thing that boys will be included in the national immunization program. If you can better protect girls by vaccinating boys, this could only be beneficial.” (F5)

On the other hand, parents addressed several potential disadvantages of the HPV vaccination for boys. Some parents mentioned concerns about the (unknown) long-term consequences and the lack of sufficient research on this. To illustrate, one parent mentioned she associated the HPV vaccine with muscle diseases and autoimmune diseases. Another parent expressed her concerns about:

“I am not sure whether it affects fertility. That would be my biggest concern. What does it do to the fertility of both the boy and the girl? Or maybe the fertility of his children? What kind of effect does it have? Or will it trigger a genetic defect somewhere, that their children will not have sperm leaders or fallopian tubes? Which could prevent them from having children at all?” (F4)

Parents who reported to be hesitant about getting their son vaccinated against HPV brought up several alternatives for vaccination. These alternatives ranged from using condoms and early testing/screening for cancers to having a healthy lifestyle (i.e. a more natural lifestyle using vitamins and having an overall healthy immune system):

“Can’t you just use condoms, similarly with AIDS and sexual transmitted diseases? Is a vaccination really needed? Or maybe education and information is sufficient?” (F4)

“I think good health comes with a good immune system. I choose for good health and good nutrition, sufficient sunlight and no excessive amount of sugar. I have good experiences with this alternative [for vaccinating].” (F9)

### Discussing vaccines and social support

The majority of the parents indicated that they could and have discussed vaccinations with people close to them. The reasons for discussing the HPV vaccination for their son with others were similar to previous vaccinations in the NIP. However, some parents addressed the desire to discuss the HPV vaccination with someone with a medical background compared to previous childhood vaccinations. Three parents indicated that they have never discussed their choices related to vaccinations and that this would not be different for the HPV vaccination for their son. One person suggested that conversations about vaccinations and the related choices were very personal and could disturb personal relationships:

“I would not discuss the decision for the HPV vaccination with people close to me. Others’ choices would make me insecure or maybe I offend others with my choices. No, this is a personal decision between my husband and I.” (F2)

### Information needs and visualizations

Most parents addressed a numerical information need related to the prevalence of HPV and the effectiveness of the HPV vaccination for boys. These informational needs could be used to evaluate the necessity of the vaccine. A need for information about the (research) development process of the HPV vaccine for boys was also mentioned. Also, parents indicated they wanted insight into the rationale for the intended lowered age at which the vaccine will be administered and the safety of the vaccine. Furthermore, some parents felt the overall risk of HPV among boys was not clear from the visuals:

“Why is the HPV vaccine offered to boys just now? What is the reason that it is needed now and that when I was young it was not there yet? Or did we not know things, that we do know now?” (M2)

### Information needs regarding the NIP

Overall, in a broader context of the provided vaccinations in the NIP, most parents indicated that the information about these vaccinations to them felt incomplete. For instance, they perceived the current information about the vaccination program to be one-sided, because counterarguments to vaccination and the importance of a healthy lifestyle are not presented. The lack of these counterarguments hindered parents in making informed decisions about vaccinations. This is also reflected in the information need parents reported regarding counterarguments for the HPV vaccination for boys. Furthermore, some parents expressed ambiguity related to the invitations they received for vaccinations in the NIP. Some parents felt that vaccination is somehow imposed, without parents being able or given the time to think about it themselves. This is related to the proposed suggestion that information about (new) vaccines should be given at an earlier moment:

“Now you get it [leaflet of the RIVM] sent along with the vaccination card. There is relatively little time or information given to make a conscious choice. It has more or less been brought up to date with the choices that your parents or your parents-in-law have made. You will base your choice on their choices. At least, if you ask me, it is no conscious choice based on the information you receive.” (F7)

## Discussion

### Main results

Overall, most parents were unaware of HPV among boys and the possibility to vaccinate against it, resulting in limited knowledge about HPV and the HPV vaccine. The majority of parents reported the presumed cross-protection for girls as the main advantage, which made it for them the main reason, to getting their son vaccinated against HPV. Several parents expressed concerns regarding unknown side-effects, in line with a previous Dutch study among Dutch parents and other international studies [15, 17, 23]. Other concerns parents shared included the need to better understand the reason to vaccinate at ten years old and objections based on religious beliefs and upbringing.

Previous studies refer to the acceptance rate of other childhood vaccinations as an important predictor for the uptake of the HPV vaccine [24, 47]. The current study also shows that parental associations and future decisions about the HPV vaccination for boys could also differ, due to the (sexual) transmissibility of HPV and its associated new scientific insights regarding the necessity of the vaccine for boys. Furthermore, the overall results stress the importance of concepts such as awareness, knowledge, and positive attitudes for acceptance of vaccination. These concepts were also found to be relevant for HPV vaccination for boys and girls in other countries [18, 19, 24]. Although the context of the Dutch NIP might differ from these countries, our results show that awareness and knowledge play an important role in the decision-making process regarding vaccination across countries. Furthermore, the current study highlights a perceived low severity of HPV in boys which could be attributed to the associations parents reported with regard to the HPV vaccination. This is in line with other studies showing that most parents associate the HPV vaccination as only available and/or useful for girls, preventing cervical cancer and/or the existing public campaign [47–52]. Finally, decisional self-efficacy seems to be important [40, 53]. The overall findings of the current study clearly show that parents expressed a need for more (balanced) information to be able to decide about HPV vaccination for their son. Finally, social support for or against HPV vaccination was not mentioned often in the current study. This might be because the vaccine for boys is not yet included in the Dutch NIP. Hence, mass media campaigns have not addressed this topic. Yet, other studies showed the importance of adhering certain social norms or consensus among parents when it concerns vaccination of their child [20, 54].

### Practical implications

It is important that the most relevant information is provided to parents through information materials as well as public campaign regarding the HPV vaccination. This includes describing the rationale of the added value of the HPV vaccination for boys in reducing HPV related cancers compared to only vaccinating girls. Equally important is including a rationale to explain the new set age at which the vaccine will be administered.

Information material regarding HPV vaccination should address the concerns parents raised in this, and several other studies, and should state whether there is scientific data to support these concerns (i.e. safety concerns regarding the HPV vaccine and misconceptions with regards to alternatives for the HPV vaccination). The latter involved parents preferring prevention of HPV infections being addressed at schools, for example by discussing the use of condoms. However, due to the transferability of HPV (e.g. skin-to-skin contact), condoms cannot provide full protection against HPV infections. Notably, debunking misconceptions is not without risks [55–57]. For example, people who at first were not doubting or thinking about these misconceptions could be triggered and become hesitant due to these misconceptions, regardless of the debunking [57]. Although emotional needs were not strongly expressed in the current study, which may be due to the approach used in our study (i.e. putting forward several topics and the presented concrete visualizations and asking questions about them during the interviews), other studies, focusing on narratives which incorporate emption do suggest their importance in effective health communication and possibly decision making about vaccination [58, 59].

In view of the broader NIP, parents stated that information about the program is sometimes sparse. Parents prefer to gain insight into the underlying (research) process when HPV vaccination for boys is added to the program. Furthermore, some parents suggested information about (new) vaccines (i.e. HPV vaccination for boys) should be given at an earlier moment. These findings could be related to low decisional self-efficacy, indicative of insufficient confidence to make informed choices, which is also identified in comparative studies [40, 53].

A Communication Activation Strategy Instrument (CASI) was applied by the National Institute for Public Health and the Environment to make sure scientific insights (about behaviour) were incorporated in communication for which one of the authors (KvZ) was also consulted [60]. The materials that were developed during this process were tested amongst several groups of parents and children, leading to a co-creation process of developing the information materials. This process hopefully leads parents to perceive information more balanced. Lastly, some parents stated that conversations with their son about the HPV vaccine would feel awkward or poses difficulties. Therefore, (referring to) information on how to discuss a topic like the HPV vaccination might increase parents’ confidence. Another suggestion would be to provide additional support of education in primary schools. This is a win-win situation as it may help in making kids more aware as well as give parents a way in to discuss the topic more openly.

### Limitations

Several factors have affected the conduct of the study. First, the COVID-19 pandemic, which emerged simultaneously to the start of the current study, affected the methodology of the study in a number of ways. Primary schools and societal organizations were closed making parents more occupied by other responsibilities, such as homeschooling children while working from home. These circumstances impeded recruitment of participants. Second, the content of the interviews was influenced by the COVID-19 pandemic, as this theme emerged often during the interviews. Some parents expressed that the current crisis emphasized the necessity and added value of vaccinations, whereas other parents expressed that the current crisis has mainly made them more aware of vaccinations. The latter made some parents skeptical and hesitant about the financial interests regarding vaccines and the underlying scientific process of ‘creating a vaccine’. The increased awareness and hesitancy regarding vaccinations, exacerbated by the search for a vaccine for COVID-19, might have influenced the results. A third limitation is the selection bias in our sample. Various participants mentioned that they have worked in healthcare organizations, which may have resulted in a sample that is not representative regarding overall perceptions of vaccination (against HPV). Moreover, most parents in the study were highly educated, which also influences the representativeness. A last limitation is the fact that we only explored parental beliefs and did not assess beliefs of boys. Their views and perceptions may also be relevant, as they also will influence interactions with parents and thus may influence parental beliefs. Therefore, additional studies among boys are recommended. Finally, although qualitative studies provide in-depth information, they do not reveal causality. Hence, quantitative longitudinal studies are recommended to analyze crucial factors related to parental decision to vaccinate their sons against HPV.

## Conclusion

The current study provides essential input for public campaigns accompanying the HPV vaccination for boys implementation in the Netherlands. Moreover, valuable lessons from our study may apply to communication regarding broader vaccination, such as NIP vaccines or COVID-19 vaccines. Unawareness amongst parents regarding the HPV vaccination for boys was high. Subsequently, there was a lack of knowledge on the how and why of the vaccination (i.e. parents felt it was mostly cross-protection of girls). Information, and communication, should not only address the unawareness and lack of knowledge, but should also take into consideration the concerns of, in this case, parents when vaccinating their child (i.e. information regarding possible adverse effects).

## Supplementary Information

**Additional file 1.**

## Data Availability

The datasets generated and/or analysed during the current study are not publicly available due to language restrictions (the interviews and transcripts are in Dutch), but are available from the corresponding author on reasonable request.

## Abbreviations

FHML REC: Faculty of Health, Medicine and Life Sciences Research Ethics Committee
GTA: Grounded Theory Approach
HPV: Human Papillomavirus
NIP: National Immunization Program
NKI: Dutch Cancer Institute
RIVM: Dutch National Institute for Public Health and the Environment
