# Supplementary material for: A qualitative study of parental associations and beliefs regarding the HPV vaccination for Dutch boys
Source: BMC Public Health. 2022 Jun 14;22:1188. doi: 10.1186/s12889-022-13605-y (PMC9196852; doi:10.1186/s12889-022-13605-y)

Visual 1. General information on the HPV vaccination

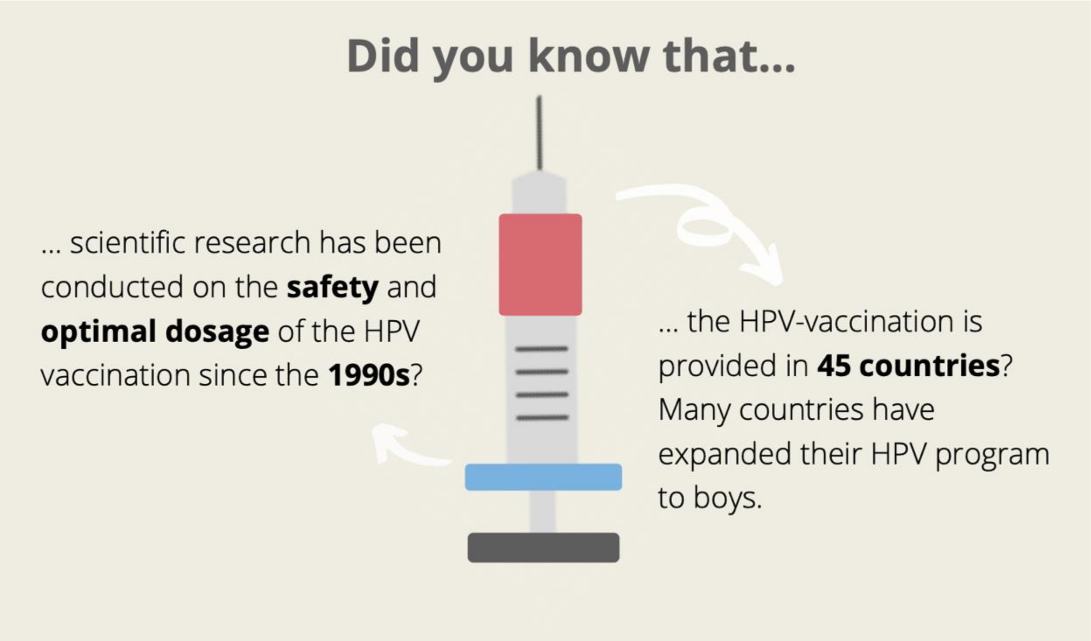

Visual 2. Clearance of HPV cells and the development of cancer

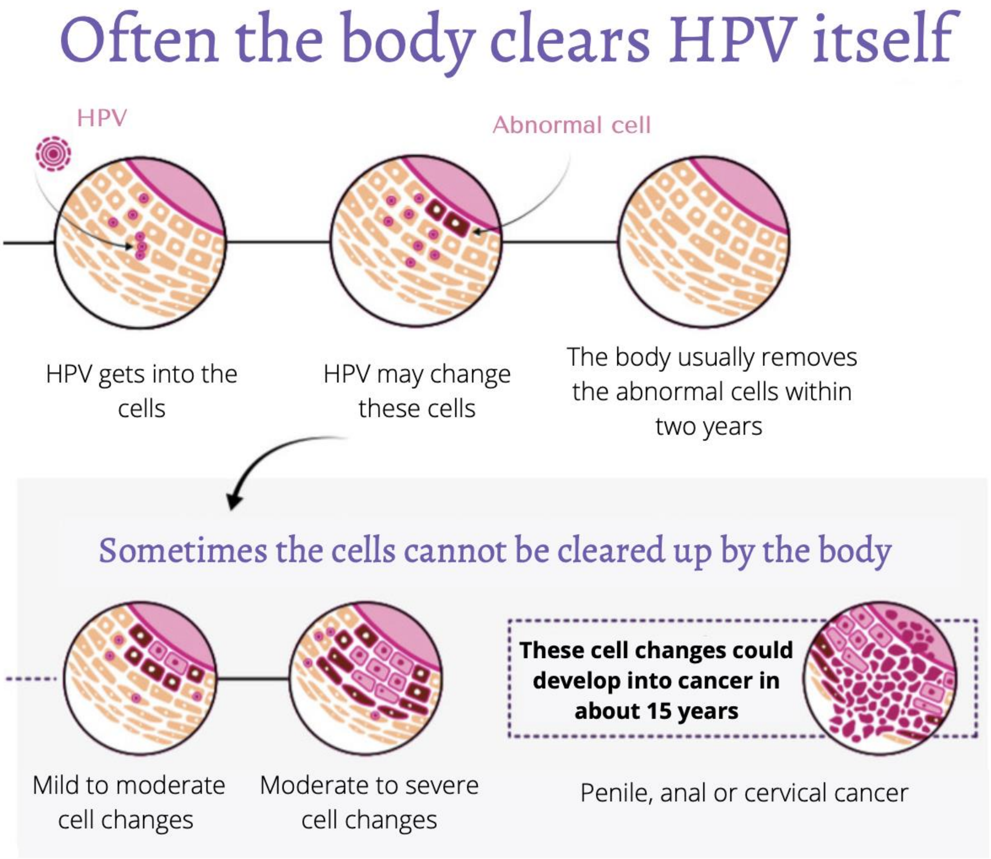

Visual 3. Physical areas of HPV associated cancers

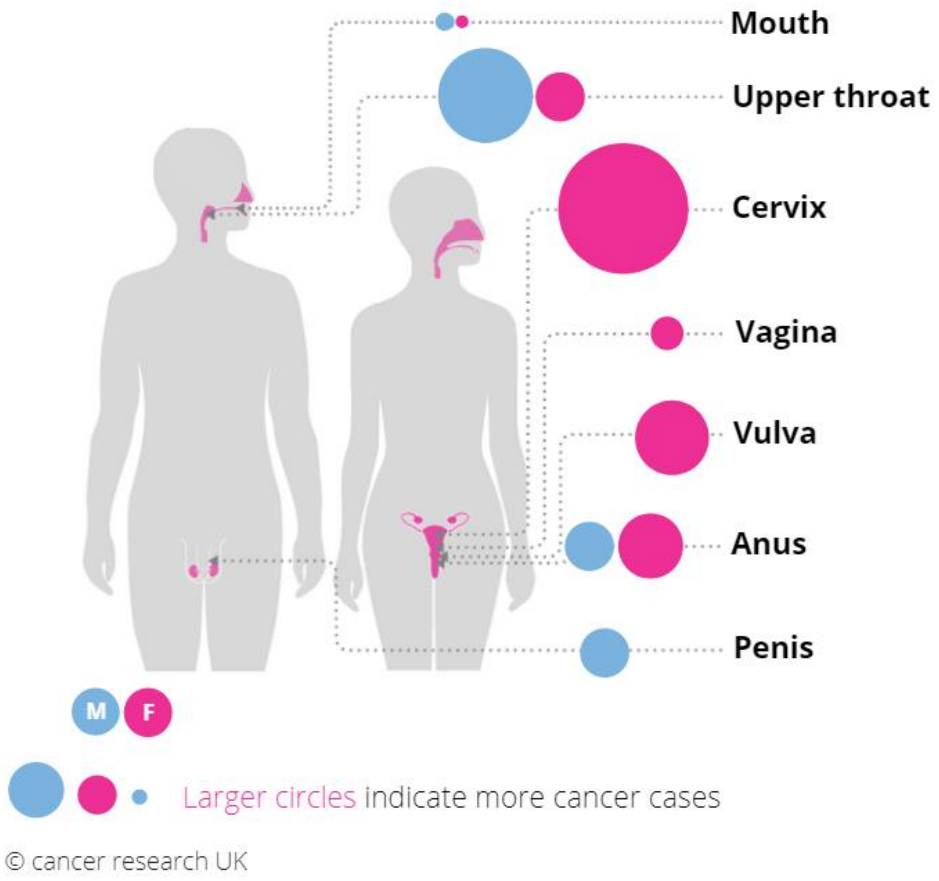

Supplement: Supplementary file 1 — Additional file 1. [file 12889_2022_13605_MOESM1_ESM.pdf]
